# Prevalence, Awareness, Treatment, and Control of Hypertension among Kazakhs with high Salt Intake in Xinjiang, China: A Community-based Cross-sectional Study

**DOI:** 10.1038/srep45547

**Published:** 2017-03-30

**Authors:** Yaoda Hu, Zixing Wang, Yuyan Wang, Lei Wang, Wei Han, Yong Tang, Fang Xue, Lei Hou, Shaohua Liang, Biao Zhang, Weizhi Wang, Kuliqian Asaiti, Haiyu Pang, Mingtao Zhang, Jingmei Jiang

**Affiliations:** 1 Department of Epidemiology and Biostatistics, Institute of Basic Medical Sciences Chinese Academy of Medical Sciences/School of Basic Medicine Peking Union Medical College, Beijing, China; 2 The People’s Hospital of Altay Prefecture, Xinjiang, China; 3 Hong Dun Town hospital, Altay Prefecture, Xinjiang, China

## Abstract

Hypertension is a leading cause of death worldwide; data on hypertension among ethnic minorities in China are sparse. This study aimed to estimate hypertension prevalence, awareness, treatment, and control in a Kazakh population, and to assess the association between salt intake and the above measures. A cross-sectional survey was conducted among Kazakh adults (≥30 years old) in the town of Hongdun, Altay, Xinjiang. Survey procedures included a questionnaire, physical measurement, and laboratory tests. Of 1805 eligible individuals, 1668 (92.4%) were included in the analysis. After adjustment for gender, age, and occupation, prevalence of hypertension was 45.5%. The proportions with awareness, treatment, control, or medication-control were 61.0%, 28.8%, 2.9% and 10.1%, respectively. Higher prevalence was seen among nomads and farmers (50.7% and 44.6%, respectively). However, the proportions with treatment or control were lower than seen among urban citizens. Hypertension prevalence was higher in those with higher salt intake (*p* = 0.0008). In contrast, the proportions with awareness (*p* = 0.0389), treatment (*p* = 0.0010), control (*p* = 0.0503), and medication-control (*p* = 0.2012) reduced as salt intake increased. In conclusion, hypertension prevalence is high in this population, but the proportions with awareness, treatment, or control are sub-optimal. Public health interventions that improve hypertension prevention and control, particularly among nomads, is needed.

Persistently elevated pressure in the arteries, also known as hypertension^1^, is a chronic, asymptomatic condition that increases an individual’s risk of developing several diseases, including coronary artery disease, stroke, and renal failure^2^. Defined by the World Health Organization (WHO) as a systolic and/or diastolic blood pressure of ≥140/90 mmHg, hypertension was the leading risk factor for global disease in 2010^3^. In 2014, over 20% of adults (≥18 years) worldwide were hypertensive and nearly 10 million deaths were caused by hypertension^4^. In China, there has been a dramatic increase in the prevalence of hypertension over the past four decades. In a representative survey conducted from 2005 to 2009 in 115 Chinese rural and urban communities involving 45108 individuals aged 35–70 years, the estimated prevalence of hypertension was up to 41.9%^5^. The 2010 Chinese guidelines for the management of hypertension^6^ point out that more than half the deaths from cardio- and cerebrovascular diseases in China are related to hypertension. The incidence of these diseases is climbing rapidly.

There is substantial evidence^7^,^8^,^9^ to show that control of hypertension can play a crucial role in preventing cardio-cerebrovascular diseases. A thorough knowledge of prevalence, awareness, treatment, and control of hypertension is needed to accomplish this pressing task. In remote, less developed areas of China, occupied mostly by ethnic minorities, patterns of hypertension may vary because of differences in economic conditions, lifestyles, and dietary habits^10^. Data on hypertension prevention and control are still relatively sparse in these individuals as compared with data on the majority Han population.

On the other hand, the association of high salt intake with hypertension have been thoroughly identified by extensive literatures. For example, a meta-analysis consisting of 18 studies in low- and middle- income countries reported odds ratios of 1.12 to 2.39 of high salt intake on prevalence of hypertension^11^. However, current data evidence is limited when it comes to the relation between salt intake and the status of awareness, treatment, and control, study of which would potentially generate practical implications for control of hypertension among populations with dietary habits of high salt intake.

In this study, we focused on Kazakh, which is a typical transnational ethnic group with a Eurasian lineage. Kazakh is the main ethnic group in Kazakhstan, and represents a major ethnic minority in China and Russia. There are approximately 1.25 million Kazakh people in China, 96% of whom reside in the remote northwest Xinjiang Autonomous Region^12^. These individuals have previously been documented to have high salt intake habits (through salted milk tea and salted air-dried beef), a high prevalence of hypertension (among the top five of the 56 ethnic groups in China), and transitional lifestyles^13^,^14^,^15^,^16^. The aims of this study were to quantify the status of hypertension prevention and control in this population, to identify influential factors and to evaluate the association, if any, between salt intake and hypertension prevalence, awareness, treatment, and control.

## Results

### Participant characteristics

A total 1805 individuals met the eligibility criteria; 1668 (92.4%) completed the survey. The mean age was 46.5 (standard deviation [SD] ± 12.3) years and 779 (46.7%) were males (Table 1). Significant differences between males and females were seen in occupation, education, BMI group, salt intake, smoking and drinking status, as well as personal history of cardiac disease. No significant difference was seen between sexes with respect to age, family history of hypertension, or annual family income.

**Figure 1:**
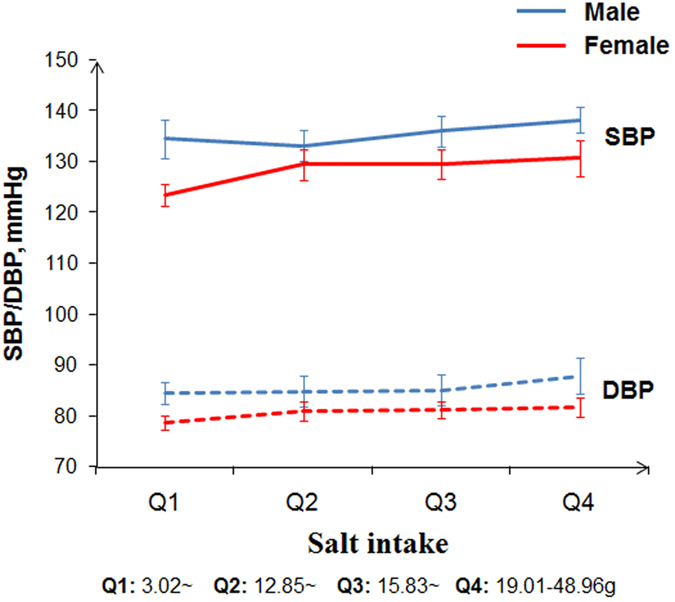
Salt intake and blood pressure for Kazakh males and females who were not taking antihypertensive drugs (n = 1450).

Among 1450 participants (86.9%) who didn’t take anti-hypertensive medication, the average blood pressure value (±SD) were: SBP135.7 (±20.6) mmHg and DBP85.7 (±12.9) mmHg for males; and SBP127.9 (±19.9) mmHg and DBP80.4 (±11.3) mmHg for females (both *p* < 0.001 by sex). The association between blood pressure and salt intake by sex is shown in Fig. 1. An increased salt intake was associated with an upward trend in SBP and DBP in males and females (all *p*_trend_ < 0.05). The SBP or DBP of males was significantly higher than that of females in different subgroups (all *p* < 0.05).

### Prevalence, awareness, treatment, control, and risk factors related to hypertension

After adjusting for gender, age, and occupation, the overall prevalence of hypertension was 45.5%. The proportions in each of the awareness, treatment, control, and medication-control groups were 61.0%, 28.8%, 2.9%, and 10.1%, respectively (Table 2). The prevalence of hypertension in males was significantly higher than in females (50.2%vs 41.3%; *p* < 0.001), but the proportion of males in the awareness (57.2% vs 65.1%), treatment (16.2% vs 42.2%), and control groups (2.1% vs 3.8%) was lower than that of females. Overall prevalence, along with the proportions in the awareness, treatment, and control groups, increased with age, but the proportion in the medication-control group showed the opposite trend (*p* < 0.05). Participants ≥70 years were most likely to have controlled hypertension, but even this proportion was less than 6%. A very low proportion of younger hypertensive patients were in the treatment group (10.2% for those aged 30–39 years). Nomads had highest overall prevalence (50.7%), with fewer individuals in the treatment and control groups. Citizens had the lowest overall prevalence (31.6%), and had more individuals in treatment and control groups. Prevalence (58.6% to 34.2%) and awareness (63.1% to 59.7%) both decreased with education, though the association was not statistically significant. Treatment, control and medication-control increased as people obtained higher education degrees (all *p*_trend_ > 0.05). Similar associations were seen with annual family income. Hypertension prevalence increased with BMI, cigarette smoking, and alcohol consumption, but no significant difference was seen in the control and medication-control groups. Those who consumed alcohol were more likely to be aware of their hypertension than those who did not (68.7% vs 59.8%, *p* < 0.001). Prevalence was higher among participants with a family history of hypertension or a personal history of heart disease, as were the proportions aware of their condition and on treatment.

Among this specific ethnic group, risk factors of hypertension with statistical significance are, males: OR = 1.56, 95% CI: (1.25–1.93); age ≥60 years: 4.83 (3.58–6.52); nomads: 1.58 (1.09–2.28) (compared with citizens); obesity: 3.63 (2.74–4.81), overweight: 1.82 (1.38–2.39) (both with normal as reference); drinking: 1.71 (1.21–2.40); family history of hypertension: 1.49 (1.18–1.87).

### Association with salt intake

**Figure 2:**
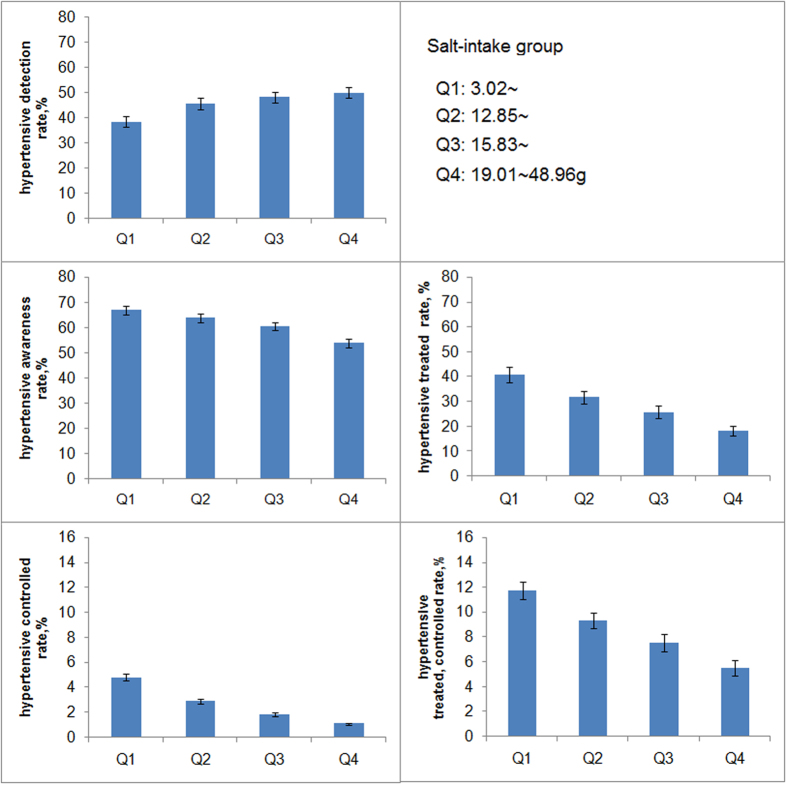
Salt intake and the proportion of individuals assigned to each of the hypertension prevalence, awareness, treatment, control, and medication-control groups. Adjusted for sex, age, and occupational background.

Further analyses of the impact of salt intake on the five measures are presented in Fig. 2. The prevalence of hypertension increased with salt intake (*p* = 0.0008); the highest prevalence was 49.9%, among individuals in the fourth quartile of salt intake (19.01–48.96 g per day). Compared with the lowest quartile of salt intake, the OR of the fourth quartile is 1.74 (1.26–2.39). The proportions in the awareness and treatment groups decreased with increasing salt intake (66.8% to 53.8% and 40.8% to 18.3%; *p* = 0.0389 and 0.001, respectively). Similar associations were seen in the control and medication-control groups, though without statistical significance (*p* = 0.0503 and 0.2012, respectively).

## Discussion

This study describes the prevalence, awareness, treatment, and control of hypertension among over 1668 Kazakh adults in Altay, Xinjiang, and estimates that 45.5% of those aged ≥30 years were hypertensive. Among those with hypertension, about two-thirds (61.0%) were aware of their condition, less than one-third (28.8%) reported taking antihypertensive medications, and very few (2.9%) had controlled blood pressure (SBP < 140 mmHg and DBP < 90 mmHg). The results of this study are similar to a cross-sectional survey conducted in Fukang pastoral area, Xinjiang, in 2008^17^. However, compared with the PURE study (participants aged 35–70 years) conducted in China^5^, individuals’ awareness was higher (61.0% vs 41.6%) and the proportions on treatment or with controlled blood pressure were lower (28.8% vs 34.4% and 2.9% vs 8.2%, respectively), suggesting that healthcare in remote areas is still sub-optimal. Higher salt intake was strongly associated with higher blood pressures and prevalence of hypertension, and negatively associated with awareness, treatment, and control.

The prevalence of hypertension, awareness, treatment, and control, when stratified by gender and age, were similar to those seen in other studies^17^,^18^,^19^. Males had higher overall prevalence, but a greater proportion of females were aware of, on treatment for, or had controlled hypertension. These differences may be explained by some of the previously observed behavioural characteristics of males in these settings, who are more likely to engage in physical labour, but less likely to be concerned about their health^19^. Overall prevalence increased with age, but more than half of those aged 30–39 years were not aware of their hypertension. In addition, the proportions receiving treatment or with controlled hypertension were below 60% and 10%, respectively, across all age groups. These were lowest among males aged 30–39 years, with only 10.2% on treatment and 1.4% with controlled hypertension. These striking figures, reinforced by other studies among Kazakhs and other ethnic minority groups^18^,^20^, highlight the urgent need for prevention work in these populations, particularly among younger men.

The prevalence of hypertension was higher among nomads and farmers, but the proportions on treatment or with controlled hypertension were lower than among citizens. These are in keeping with findings from studies of other ethnic groups, which compared measures between rural and urban areas^5^,^9^,^18^,^21^. Over half the nomads in the sample had hypertension, which may be explained by the long-term physical labour in animal husbandry and their dietary habits (more than 90% of nomads drink milk tea, and vegetables and fruits are not a staple). The differences in the proportions on treatment may have been influenced by the annual family income and levels of education: over half the nomads and farmers had an annual family income below 10 000 RMB, in contrast to less than 20% of citizens; and the proportions with middle-school education or higher were 9.9% and 20.1% for nomads and farmers, respectively, compared with 90.7% among citizens. This suggests a need to improve education and economic prospects of nomads and farmers to achieve more effective control of hypertension, although the lifestyle of most nomads (who have no permanent residence) may impede effective monitoring of medication compliance. The use of special approaches, such as portable medication guides, should be explored among these individuals.

Several studies^19^,^22^,^23^,^24^ have shown that hypertension prevalence, as well as the proportions aware and on treatment were higher among those with established risk factors, such as obesity, alcohol consumption, a family history of hypertension, or a history of heart disease. This is thought to be because people with the above risk factors may be more concerned about their health and are more likely to accept antihypertensive treatment to avoid cardiovascular or other adverse events. Results from this study were similar, other than among smokers, fewer of whom were aware or on treatment compared with non-smokers. Further analysis suggests this was because the smokers sampled were mostly young people, who had less knowledge about their hypertension and were less willing to be treated.

In particular, this study explored relationships between hypertension awareness, treatment, control, and medication-control and levels of salt intake, a well-established risk-factor for hypertension^25^,^26^. The proportions with awareness or on treatment decreased as salt intake increased. This may be because limiting salt intake has become an important lifestyle intervention in the prevention and control of hypertension and may lead to patients changing their dietary habits. Salt intake was significantly lower in patients with more than 2 years of hypertension compared with those with less than 2 years’ history (15.81 g vs17.57 g per day). Hypertensive participants with lower salt intake were more likely to be controlled or medication-controlled, indicating that the hypertension seen in this population is highly salt-sensitive and the salt intake may have an impact on the efficacy of antihypertensive drugs. This was further suggested by the data that blood pressures increased with salt intake among those on antihypertensive treatment, while no differences were seen among those with hypertension but not on treatment. Aware of the harmful impact of salt-intake on hypertension-related health outcomes (e.g., a daily salt intake of 7.0 g and more, compared with 4.00–5.99 g increased 25% deaths and 16% cardiovascular events in an international cohort study covering more than 150,000 participants^27^), our results can also provide reference to other ethnic minorities with dietary habit of high salt intake such as Tibetan and Mongolian in China, and to other parts of the world such as the south-east and central Asia, parts of Europe, and northwest Africa, whose mean intake of salt is more than 10 g/day according to global status report on noncommunicable diseases 2014^4^.

This study had limitations: the strength of the observed associations is limited as a result of the cross-sectional study data, with data on exposures and outcomes collected at the same time, making it difficult to explore any temporal changes in outcomes. The sample size was too small to obtain stable estimates in some subgroup analyses, particularly among citizens and among those with medication-controlled hypertension. Besides, we used the second urine sample after waking to estimate individual salt intake instead of 24-h urine collection for convenience (especially among the nomads), therefore a slight measurement error may exist though the second urine sample was thought as a satisfactory surrogate for epidemiological surveys^27^,^28^. Finally, health-care service coverage may have an influence on the relationship of salt intake with hypertension awareness and treatment, and participants were asked only if they were taking antihypertensive drugs and not about the names or doses of drugs, limiting the ability to evaluate the low proportion with medication-controlled hypertension in this population. A key strength of this study is the high-quality study design and implementation with a high response rate, which helped to ensure internal validity and allows for extrapolation of study results. Further studies are needed to provide more detailed information to better guide hypertension control in this ethnic population.

## Conclusion

In conclusion, the prevalence of hypertension is high among Kazakhs living in Altay, Xinjiang, China. The proportions of individuals who are aware of, on treatment for, or have controlled disease are far from ideal. It is suggested that the following measures be taken to improve hypertension prevention and control: (1) establish blood pressure measurement points, so residents may improve their awareness; (2) implement initiatives at an administrative village level to reduce salt intake, as the high intake has a significant impact on prevalence and control of hypertension; and (3) pay special attention to certain subgroups in conducting prevention work, particularly males, younger individuals, and nomad populations.

## Methods

### Study population

The survey was conducted in the town of Hongdun, a rural–urban continuum area of the Altay in the north of Xinjiang. This area was chosen because it is a Kazakh-concentrated area that is representative of Altay’s economy, according to local financial data; the Kazakhs living there are representative of this ethnic group, as interracial marriage is uncommon, reducing the chances of genetic heterogeneity affecting estimations; and the rural–urban continuum area may help to explore the influence of lifestyles, as the Kazakhs there naturally form three occupational groups: nomads, farmers, and citizens. Twelve administrative villages and one township office were initially included after excluding villages with fewer than 100 Kazakh people living with other ethnic groups. Stratified random cluster sampling was performed; the final sample included two pastoral villages, three agricultural villages, and one township office.

In sampled units, all Kazakh people aged ≥30 years were recruited. Pregnant women, bedridden or disabled persons, and individuals with mental disorders or other severe disease (as determined by doctors) were excluded. Detailed inclusion and exclusion criteria have previously been reported^29^. Propaganda measures such as posters and household publicity were performed in each survey spot and during recruitment process, and lists of attendance status were on a daily basis fed back to the principals of the sampled units, who coordinated to guarantee high response rate.

### Ethnic statement

The study was approved by the Ethics Committee of the Institute of Basic Medical Sciences of the Chinese Academy of Medical Sciences, and written informed consent was obtained from all participants. The study was performed in accordance with approved national guidelines.

### Survey method

Baseline data were collected between October 2012 and February 2013. Participants were invited to take a face-to-face questionnaire and to be physically measured. A unified questionnaire was used that included questions on demographics, socioeconomic status (educational level and annual family income), cigarette smoking, alcohol consumption, and information about personal or family history of selected conditions. Individuals were categorised into never- and ever-smokers; the latter category included current and former smokers. Participants were similarly classified as never- and ever-drinkers with regard to alcohol consumption.

Physical measurements were obtained by trained and certified staff using standard protocols. Body weight was measured to the nearest 0.01 kg; subjects were weighed without shoes and in minimal clothing. Height was measured to the nearest 0.001 m and body mass index (BMI; kg/m^2^) was calculated. In line with Chinese Obesity Working Group recommendations, individuals with a BMI of 24–27.9 kg/m^2^ were considered overweight, and those with a BMI ≥ 28 kg/m^2^ were considered obese^30^. Seated right brachial blood pressure (mmHg) was measured twice, using a mercury sphygmomanometer after a rest period of at least 10 minutes. A third measurement was taken if a difference of ≥5 mmHg was observed between the first and second measurements. The final reading was the mean of all two or three measurements.

Besides, second early-morning urine sample was collected from each participant. Samples were refrigerated immediately and transported in cold storage to the central laboratory of the People’s Hospital of Altay. These samples were used to estimate sodium intake by measuring urinary sodium excretion; urinary creatinine concentration, and 24-hour urinary creatinine excreted estimated from height, body weight, and age^31^. Daily salt intake was estimated based on the assumption that all sodium ingested was in the form of sodium chloride (*salt*), with each 43 mmol of sodium being equivalent to approximately 2.5 g of salt. Electrolytes were measured using a Caretium XI-921 CT Electrolyte Analyzer (Shenzhen, China).

### Classification criteria

Hypertension was defined as a systolic blood pressure (SBP) ≥ 140 mmHg or diastolic blood pressure (DBP) ≥ 90 mmHg at the time of measurement, or as having taken antihypertensive medication in the recent 2 weeks. *Hypertension awareness* was defined as the proportion of hypertensive participants who self-reported having been diagnosed with hypertension. *Treatment* was defined as the proportion of hypertensive participants taking antihypertensive drugs in the recent 2 weeks. *Controlled* refers to the proportion of hypertensive participants with SBP < 140 mmHg and DBP < 90 mmHg at the time of measurement. *Medication-controlled* refers to the proportion of participants taking antihypertensive drugs with SBP < 140 mmHg and DBP < 90 mmHg at the time of measurement.

### Statistical analysis

Differences between means or medians of quantitative measures were tested using Student’s t-test or the Mann–Whitney U test. Qualitative data were expressed as proportions; testing for differences was performed using a chi-square test. Logistic regression models were used to estimate prevalence, awareness, treatment, control, and medication-control of hypertension with 95% confidence intervals (CI) after adjusting for gender, age, and occupation. A generalized linear model, stratified by sex, was used to test for associations between SBP or DBP and salt intake. All analyses were performed with Statistical Analysis System 9.2 (SAS Institute Inc., Cary, NC, USA); a *p*-value < 0.05 was considered statistically significant.

## Additional Information

**How to cite this article**: Hu, Y. *et al*. Prevalence, Awareness, Treatment, and Control of Hypertension among Kazakhs with high Salt Intake in Xinjiang, China: A Community-based Cross-sectional Study. *Sci. Rep.* **7**, 45547; doi: 10.1038/srep45547 (2017).

**Publisher's note:** Springer Nature remains neutral with regard to jurisdictional claims in published maps and institutional affiliations.

## Figures and Tables

**Table 1:** Characteristics of the participants.

	All (n = 1668)	Male (n = 779)	Female (n = 889)	P
Age (years), Mean ± SD	46.5 ± 12.3	46.5 ± 12.1	46.6 ± 12.5	0.8934
Age (years) group, (%)				0.5368
30~	35.7	35.3	36.1	
40~	27.9	29.0	26.6	
50~	19.8	18.6	21.2	
60~	11.3	11.3	11.3	
70~	5.4	5.9	4.9	
Occupation, (%)				0.0063
Nomad	38.2	38.4	38.0	
Farmer	50.2	52.6	48.1	
Citizen	11.6	9.0	13.8	
Education attainment, (%)				0.0010
Primary school or below	30.3	30.8	29.8	
Middle school	45.3	48.8	42.2	
High school and above	24.4	20.4	28.0	
Family annual income (RMB), (%)				0.6685
<10 000	53.2	52.0	54.2	
10 000–39999	41.2	42.0	40.4	
≥40 000	5.7	6.0	5.5	
BMI group, (%)*				<0.0001
Normal	35.5	40.0	31.6	
Overweight	32.0	33.7	30.5	
Obesity	32.5	26.3	37.8	
Salt intake (g), Median (IQR)	16.5 (12.9–19.0)	17.5 (13.8–20.1)	15.7 (12.1–17.9)	<0.0001
Smoking, (%)				<0.0001
Yes	35.9	73.0	3.5	
No	64.1	27.0	96.5	
Drinking, (%)				<0.0001
Yes	13.4	28.4	0.2	
No	86.6	71.6	99.8	
Family history of hypertension, (%)				0.5303
Yes	55.3	54.2	56.2	
No	40.6	41.2	40.1	
Unknown	4.2	4.7	3.7	
Personal history of Cardiac disease, (%)				<0.0001
Yes	12.8	7.6	17.4	
No	87.2	92.4	82.6	

BMI: body mass index; RMB: renminbi; SD: standard deviation. *BMI groups: Normal < 24 kg/m^2^; Overweight 24–27.9 kg/m^2^; Obese ≥ 28 kg/m^2^.

**Table 2:** Factors associated with prevalence, awareness, treatment, controlled, and medication-controlled of hypertension, %*.

Variables	Prevalence (95% CI)	Awareness (95% CI)	Treatment (95% CI)	Controlled (95% CI)	Medication-controlled (95% CI)
Sex					
Male	50.2 (48.7–51.7)^**‡**^	57.2 (56.0–58.4)	16.2 (15.1–17.2)^**‡**^	2.1 (1.9–2.2)	12.7 (12.2–13.2)
Female	41.3 (39.9–42.7)	65.1 (64.0–66.3)	42.2 (40.6–43.9)	3.8 (3.6–4.0)	9.0 (8.8–9.3)
Age group (years)					
30~	23.6 (23.1–24.0)^**‡**^	42.0 (41.6–42.4)^**‡**^	10.2 (9.0–11.4)^**‡**^	1.4 (1.3–1.6)	12.3 (10.3–14.2)
40~	41.0 (40.4–41.7)	53.5 (53.1–53.8)	18.7 (17.2–20.1)	2.1 (1.9–2.2)	11.4 (10.6–12.2)
50~	61.0 (60.3–61.8)	64.2 (63.9–64.5)	30.0 (28.3–31.7)	2.9 (2.8–3.1)	10.5 (9.9–11.1)
60~	77.6 (76.9–78.4)	73.6 (73.3–73.9)	42.5 (40.0–45.2)	3.9 (3.7–4.2)	9.4 (9.0–9.8)
70~	88.6 (88.0–89.2)	81.3 (81.0–81.6)	58.8 (55.5–62.1)	5.7 (5.3–6.1)	8.6 (7.9–9.3)
Occupation					
Nomad	50.7 (49.1–52.3)^**‡**^	60.7 (59.3–62.0)	24.8 (22.9–26.6)^**‡**^	2.2 (2.0–2.3)	8.8 (8.4–9.1)
Farmer	44.6 (43.2–46.1)	62.7 (61.5–63.9)	31.4 (29.4–33.3)	3.2 (3.1–3.4)	10.3 (9.9–10.6)
Citizen	31.6 (29.2–34.0)	52.5 (49.5–55.4)	34.4 (29.5–39.3)	4.9 (4.3–5.5)	14.3 (13.3–15.3)
Education attainment					
Primary school or below	58.6 (56.7–60.5)	63.1 (61.4–64.8)	33.0 (30.5–35.4)	2.9 (2.7–3.0)	8.6 (8.2–8.9)
Middle school	42.5 (41.1–43.9)	60.5 (59.2–61.8)	24.9 (23.1–26.7)	2.6 (2.4–2.7)	10.5 (10.0–10.9)
High school and above	34.2 (32.5–35.9)	59.7 (57.3–62.0)	30.3 (27.1–33.4)	3.9 (3.5–4.3)	12.9 (12.1–13.7)
Family annual income (RMB)					
<10 000	47.7 (46.2–49.1)	64.8 (63.6–65.9)^**‡**^	28.2 (26.4–30.0)	2.0 (1.9–2.2)	6.9 (6.7–7.1)
10 000–39999	43.2 (41.6–44.9)	57.8 (56.3–59.3)	29.4 (27.3–31.6)	3.8 (3.6–4.0)	13.5 (12.9–14.0)
≥40 000	37.5 (33.2–41.7)	47.3 (42.9–51.7)	28.0 (22.0–34.0)	6.5 (5.2–7.8)	23.1 (20.5–25.7)
BMI group^†^					
Normal	32.9 (31.1–34.7)^**‡**^	58.2 (56.3–60.0)	23.2 (20.8–25.6)	2.2 (2.0–2.4)	9.4 (8.9–10.0)
Overweight	45.2 (43.4–47.0)	59.9 (58.4–61.4)	26.6 (24.4–28.9)	2.6 (2.5–2.8)	10.2 (9.5–10.8)
Obese	59.3 (57.5–61.1)	63.7 (62.3–65.0)	33.7 (31.5–35.8)	3.5 (3.3–3.7)	10.4 (9.9–10.9)
Smoking					
Yes	46.4 (44.7–48.1)^**‡**^	57.8 (56.3–59.2)	20.2 (18.5–22.0)^**‡**^	3.2 (3.0–3.5)	16.1 (15.4–16.7)
No	44.7 (43.4–46.1)	63.4 (62.3–64.6)	34.0 (32.2–35.8)	2.7 (2.6–2.9)	8.0 (7.8–8.2)
Drinking					
Yes	59.0 (56.6–61.4)^**‡**^	68.7 (66.8–70.6)^**‡**^	15.3 (13.5–17.0)^**‡**^	2.3 (2.1–2.5)	15.0 (14.0–16.0)
No	43.2 (42.1–44.4)	59.8 (58.6–61.0)	31.8 (30.6–33.1)	3.1 (2.9–3.2)	9.6 (9.3–10.0)
Family history of hypertension					
Yes	44.4 (43.2–45.7)^**‡**^	65.7 (64.5–66.9)^**‡**^	32.1 (30.1–34.1)^**‡**^	3.2 (3.0–3.4)	9.9 (9.5–10.3)
No	43.6 (41.9–45.4)	54.1 (52.3–55.9)	21.8 (19.8–23.8)	2.7 (2.5–2.9)	12.5 (11.8–13.2)
Unknown	73.9 (69.0–78.8)	68.6 (64.5–72.8)	45.1 (38.3–51.9)	2.0 (1.6–2.3)	4.4 (3.9–4.8)
Personal history of cardiac disease					
Yes	63.8 (61.2–66.5)	78.7 (77.3–80.1)^**‡**^	55.9 (52.7–59.1)^**‡**^	6.6 (6.1–7.1)^**‡**^	11.8 (11.1–12.6)
No	42.6 (41.5–43.7)	57.5 (56.6–58.5)	23.0 (21.8–24.2)	2.1 (2.0–2.2)	9.2 (8.7–9.6)

^*^Adjusted for sex, age, and occupational background; ^†^BMI: body mass index. Normal: BMI < 24 kg/m^2^; Overweight: 24 ≤ BMI < 27.9 kg/m^2^; Obesity: BMI ≥ 28 kg/m^2^; ^‡^*p* < 0.05.
